# A Case of Liver Metastasis from Small Intestinal Gastrointestinal Stromal Tumor 25 Years after Surgery including Autopsy Findings

**DOI:** 10.1155/2021/6642427

**Published:** 2021-02-24

**Authors:** Yuichi Takano, Masataka Yamawaki, Jun Noda, Tetsushi Azami, Takahiro Kobayashi, Fumitaka Niiya, Eiichi Yamamura, Naotaka Maruoka, Kuniyo Gomi, Yuichiro Kuroki, Yorimasa Yamamoto, Tomoko Norose, Nobuyuki Ohike, Masatsugu Nagahama

**Affiliations:** ^1^Division of Gastroenterology, Department of Internal Medicine, Showa University Fujigaoka Hospital, Yokohama, Kanagawa, Japan; ^2^Yamamura Clinic, Matsumoto, Nagano, Japan; ^3^Department of Diagnostic Pathology, Showa University Fujigaoka Hospital, Yokohama, Kanagawa, Japan

## Abstract

Gastrointestinal stromal tumor (GIST) is the most common mesenchymal tumor in the digestive tract. Recurrences may occur even after radical resection; however, recurrence later than 10 years after surgery is rare. We report a case of GIST with recurrence of liver metastasis 25 years after surgery. A 56-year-old man complained of sudden epigastric pain and was transferred to the emergency department. He had undergone partial resection of the small intestine for leiomyosarcoma 25 years previously. Abdominal computed tomography showed multiple liver tumors with massive hemorrhage. Ultrasound-guided percutaneous biopsy was performed for the 15-mm hepatic tumor in segment 2. Pathological findings revealed proliferation of spindle-shaped atypical cells, and immunostaining for c-kit and CD34 was both positive; the patient was therefore diagnosed with GIST. He then underwent chemotherapy for 7 years but died of multiple organ failure due to GIST. Autopsy revealed GIST occupying the entire liver with peritoneal dissemination, and minute lung metastases that could not be identified by CT were also detected. This case is interesting in considering the recurrence of GIST, and we will report it together with the literature review.

## 1. Introduction

Gastrointestinal stromal tumor (GIST) is the most common mesenchymal tumor in the digestive tract. Resection is typically the first treatment choice because of its high potential for malignancy. Recurrences may occur even after radical resection; however, recurrence later than 10 years after surgery is rare [1, 2]. We report a case of GIST with recurrence of liver metastasis 25 years after surgery together with autopsy findings.

## 2. Case Presentation

A 56-year-old man complained of sudden epigastric pain and was transferred to the emergency department. He had undergone partial resection of the small intestine for leiomyosarcoma 25 years previously at another hospital (31-year-old). He had an unremarkable family history, except for a father with aplastic anemia. He consumed alcohol occasionally and had never smoked.

On admission, vital signs included a blood pressure of 108/65 mmHg, a pulse rate of 112 beats/min, a respiratory rate of 12 breaths/minute, and an oxygen saturation of peripheral artery (SpO_2_) 98%. Tenderness was noted in the epigastric region, but no signs of peritoneal irritation were seen. Diminished peristaltic sounds were noted. Blood tests showed a total bilirubin of 3.9 mg/dL, aspartate transaminase (AST) of 283 U/L, alanine aminotransferase (ALT) of 301 U/L, alkaline phosphatase (ALP) of 1400 U/L, and gamma-glutamyl transpeptidase (*γ*GTP) of 324 U/L, indicating hepatobiliary dysfunction, with a hemoglobin (Hb) level of 10.2 g/dL, suggestive of anemia. Tumor markers (alpha-fetoprotein [AFP], protein induced by vitamin K absence or antagonist-II [PIVKA-II], carcinoembryonic antigen [CEA], and carbohydrate antigen 19-9 [CA19-9]) were all within the normal range. The patient was negative for hepatitis C virus antibodies (HCVAb) and hepatitis B surface antigen (HBsAg).

**Figure 1:**
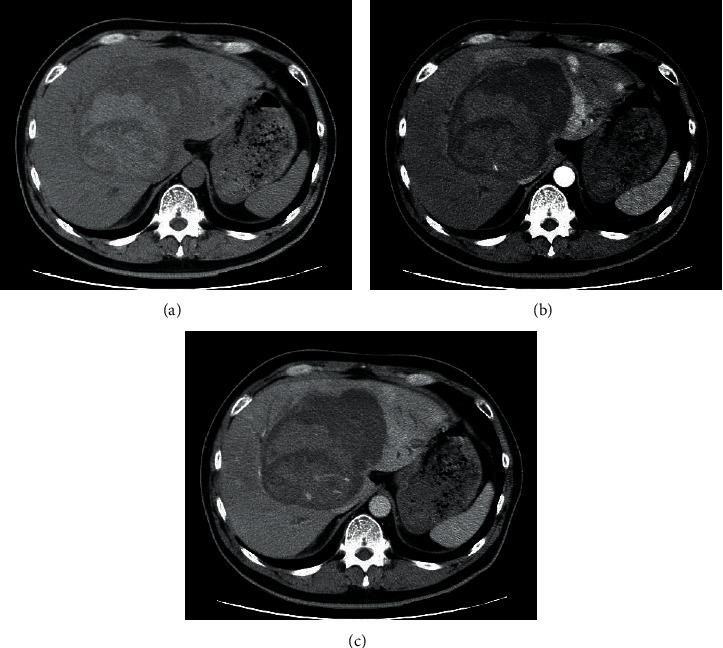
(a) Plain abdominal CT showing an irregular high-density area in the center of the liver and bleeding is suspected. Tumor detection is difficult. (b) Contrast-enhanced CT (arterial phase) revealing multiple tumors with a contrast effect in the left lobe of the liver (arrow). The patient was diagnosed with tumor-associated hemorrhage. (c) Abdominal CT (late phase) disclosing that the tumor exhibits the same contrast effect as normal liver tissue.

**Figure 2:**
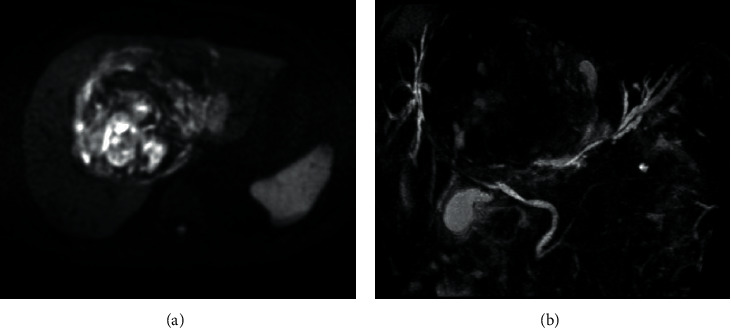
(a) Diffusion-weighted MRI images showing multiple tumors with diffusion decrease in the left lobe of the liver. (b) On MRCP, the intrahepatic bile duct was observed to be markedly compressed by bleeding in the hilum of the liver.

Abdominal computed tomography (CT) showed an irregular high-density lesion in the center of the liver, and bleeding was suspected. A multiple mass with contrast effects was observed in the left lobe of the liver, which was considered to be a tumor-associated hemorrhage (Figure 1). Diffusion-weighted magnetic resonance imaging (DWI-MRI) of the abdomen also revealed multiple tumors with reduced diffusion in the left lobe. On magnetic resonance cholangiopancreatography (MRCP), the intrahepatic bile duct was markedly compressed due to the bleeding (Figure 2). Upper and lower endoscopy and capsule endoscopy were performed, but no neoplastic lesion including submucosal tumor was found in the digestive tract.

**Figure 3:**
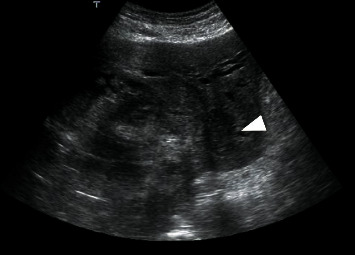
Abdominal ultrasonography showing a 15-mm hypoechoic mass in liver segment S2 adjacent to the bleeding (arrowhead). A percutaneous biopsy was performed.

**Figure 4:**
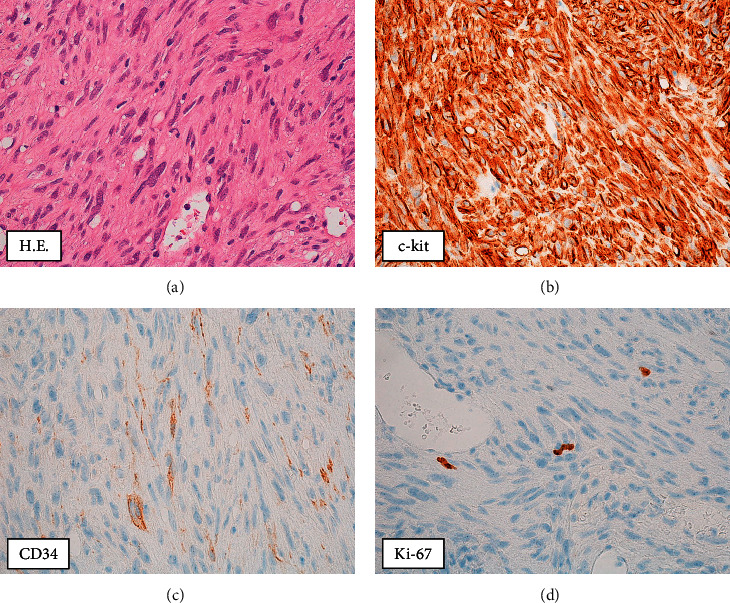
Pathological analysis of the liver biopsy revealed dense proliferation of spindle-shaped atypical cells. The tumor cells were positive for c-kit and CD34, and the patient was diagnosed with GIST. The MIB-1 labelling index was 2% (x 400).

As the central part of the liver was diagnosed with a giant hemorrhage from tumor, ultrasound-guided percutaneous biopsy was performed for the 15-mm hepatic tumor in segment 2 (Figure 3). Pathological findings revealed proliferation of spindle-shaped atypical cells, and immunostaining for c-kit and CD34 were both positive; the patient was therefore diagnosed with GIST. The MIB-1 labelling index was 2% (Figure 4). The atypical mitotic figure was 2/50 high-power fields (HPF). A mutation was found in Exon 11 in the C-kit gene search. Since the last operation was 25 years ago, the case was closed, and no pathological specimens from that time were available for analysis. The size of the small intestinal tumor was unknown, because his medical record was not available. However, based on patient history and clinical course, the patient was diagnosed with GIST with metastasis to the liver 25 years after the initial surgery.

Imatinib (400 mg/day) was introduced; however, 8 days after the initiation, the patient complained of sudden abdominal pain. Abdominal CT revealed intra-abdominal bleeding due to massive bleeding from the GIST liver metastases. Emergency transcatheter arterial embolization (TAE) was performed, and hemostasis was achieved.

We considered the possibility that Imatinib might have triggered the tumor bleeding. However, since the mutation in Exon 11 is a high-response marker for imatinib, the drug was reintroduced after informed consent. Ten days after treatment re-initiation, intra-abdominal hemorrhage was observed again. Because of acute renal failure, the patient was treated for 6 days in the intensive care unit. It was decided that imatinib could not be continued, and the patient was therefore started on sunitinib after improvement of his general condition (57-year-old).

**Figure 5:**
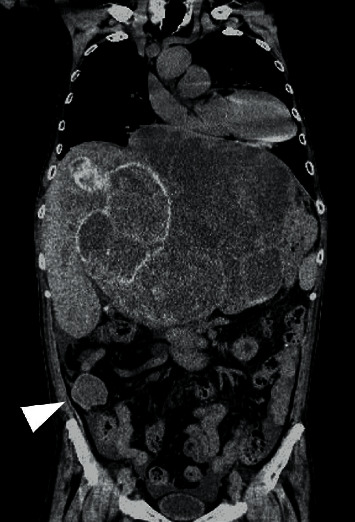
CT findings one month before death. Multiple metastases of GIST are found in the liver. A 40 mm nodule is found in the ileocecal region (arrowhead). No tumor was found in both lungs.

Because of hand-foot syndrome, liver dysfunction, and cytopenia, sunitinib (50 mg/day) was administered for 1 week, followed by 2–4 weeks off treatment. Stable disease (SD) was maintained until the 50th course, and no bleeding complication was observed. However, progressive disease (PD) was observed when evaluating CT images after 58 courses (liver metastases were clearly increased in size), and we switched the patient to regorafenib (120 mg/day), which is the third line of chemotherapy (62-year-old). The patient achieved SD after 10 courses of regorafenib therapy for one week and resting for 2–4 weeks. However, CT imaging revealed apparent PD after 19 courses (liver metastasis increased rapidly, although no metastases were seen in other organs), the chemotherapy was discontinued, and palliative treatment was administered for next one year. Seven years after the diagnosis of liver metastasis, he died of multiple organ failure due to GIST (Figure 5) (63-years-old).

**Figure 6:**
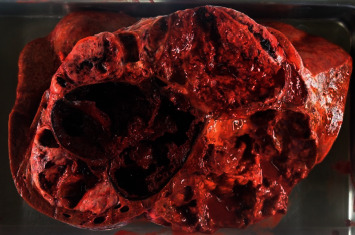
On autopsy, a tumor with necrotic hemorrhage extending throughout the liver could be seen macroscopically, with only little normal liver tissues remaining.

**Figure 7:**
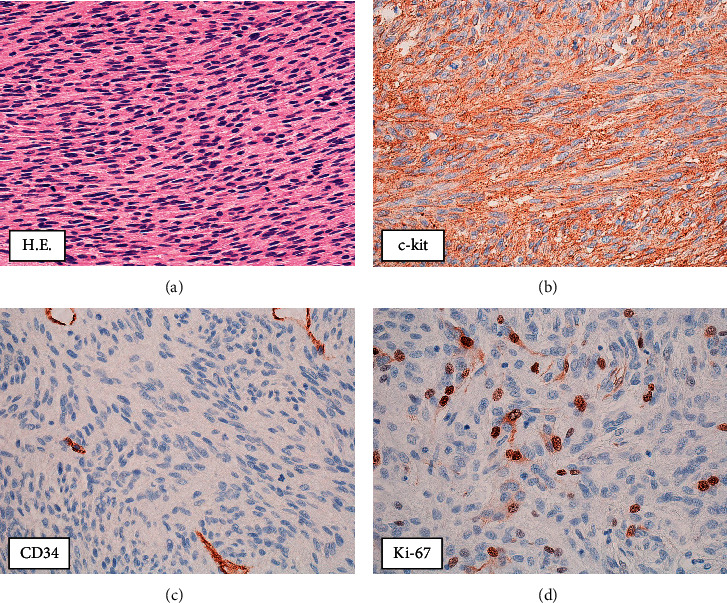
Pathological findings in the liver tumor at autopsy. Proliferation of atypical spindle-shaped cells was observed. The tumor was c-kit- and CD34-positive, confirming the diagnosis of GIST. MIB-1 labelling index was 12% (x 400).

**Figure 8:**
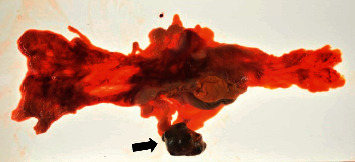
On autopsy, a 40-mm nodule was found in the ileocecal region, and the pathological finding was compatible with GIST. The patient was diagnosed with peritoneal dissemination of GIST.

**Figure 9:**
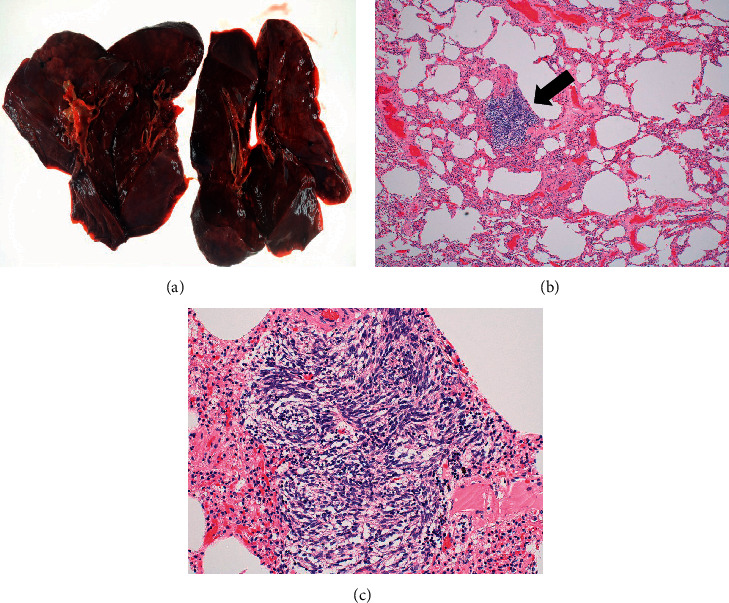
Lung findings at autopsy. (a) Macroscopic findings did not indicate a clear tumor in the lungs (x100). (b, c) Microscopically, multiple small lung metastases of GIST were observed in all lung lobes (arrow) (x200, Hematoxylin-Eosin stain).

Autopsy was performed with the consent of his family. The liver weighed 6,300 g and was markedly swollen. Macroscopically, a tumor mass with hemorrhagic necrosis was found throughout the liver, with only a small amount of normal liver tissue remaining (Figure 6). Pathologically, spindle-shaped tumor cells similar to those observed on liver biopsy were observed. The tumor cells were c-kit- and CD34-positive, and the diagnosis of GIST was confirmed. The MIB-1 labelling index was 13%, and the atypical mitotic figure was 13/50 HPF (Figure 7). A 4-cm tumor was found on the terminal ileal serosa. Pathologically, it was diagnosed with peritoneal dissemination of GIST (Figure 8). Although macroscopic findings of lungs did not indicate a clear tumor, multiple minute lung metastases of GIST were observed in all lung lobes microscopically (Figure 9). No metastases were observed in other organs both macroscopically and microscopically.

## 3. Discussion

GIST originates from interstitial cells of Cajal in the muscular layer of the digestive tract and is caused by mutations in the c-kit and PDGFRA genes [2, 3]. This neoplasm comprises about 80% of all gastrointestinal mesenchymal tumors. It occurs in 2/100,000 individuals and is most common in their 50s and 60s. Reportedly, the most common primary site is the stomach (60%–70% of the cases), while 20%–30% of the cases are located in the small intestine, with 5% of the cases located in the large intestine or esophagus [4, 5]. Due to its malignant nature, resection is the first choice. However, even in radically resected cases, recurrence is observed in about 40% of the cases [1]. The recurrence type is local recurrence (33%), metastasis recurrence (48%), or local + metastasis recurrence (19%). In case of recurrence with metastasis, the liver is the most frequent site of metastasis [1, 2]. The risk of recurrence depends on tumor size, mitotic figures, and primary site, and various risk classifications have been reported [6, 7]. According to the classification by Miettinen et al., the recurrence rate is 0%–8% in those with “very-low to low risk”, 10%–24% in those with “moderate risk”, and 34%–90% in those with “high risk” [7]. Most postoperative recurrences occur within 2 years, whereas recurrence after 10 years or more is considered rare [2].

The present patient had undergone small intestinal resection 25 years previously with a diagnosis of leiomyosarcoma. At that time, GIST had not been recognized as a disease entity, and a definite diagnosis could not be made. The diagnosis of GIST was established retrospectively based on the clinical course and the finding of liver metastases 25 years after surgery. Unfortunately, there were no pathological specimens from the time of the initial surgery, and so evaluation of the original tumor size and mitotic figures was not possible.

We performed a literature search in PubMed on cases of GIST recurring 10 years or more after surgery, using the keywords “GIST, metastasis” between 1995 and 2019 (English literature only). Six cases including our case were reported (Table 1) [8–12]. The average age of these six cases was 56 years (range, 39–71), and in these four male and two female patients, the primary tumor site was either stomach (*n* = 1), duodenum (*n* = 1), small intestine (*n* = 3), or rectum (*n* = 1), and the average tumor size was 6.9 cm (range, 2.5–14.0). The mitotic index was <5/50 HPF in the three cases for which information on mitotic index was available, and according to the risk classification by Flecher, two patients were considered “low”, one was classified as “intermediate”, one as “high”, and for one patient, the risk status could not be determined. The average time to recurrence was 18.5 years (range, 11–25).

Cases recurring more than 10 years after surgery included low-to high-risk patients according to the Flecher classification [6]. Thus, conventional risk classification was not effective to predict recurrence after a long time. On the other hand, focusing on the mitotic index, it can be deduced that all the three cases with mitotic index data had less than 5/50 HPF. In our case, the liver biopsy obtained at the time of recurrence had a MIB-1 labelling index of 2% and a mitotic index of 2/50 HPF, suggesting a tumor with low proliferative ability. We speculate that low-malignant tumors are characterized by microscopic pathology (atypical mitotic figure <5/50 HPF) and develop very slowly, which may lead to recurrence after a long time. Pidhorecky et al. also reported that recurrence long after the operation could be seen in patients with GIST with low atypical mitotic figures [13]. The current guidelines recommend follow-up for up to 10 years after surgery [2], but it should be noted that relapse may occur later than 10 years after surgery. For pathologically low-grade GIST (atypical mitotic figure <5/50 HPF), long-term follow-up for more than 10 years is recommended.

In the present case, minute lung metastases were found during autopsy. These pulmonary metastases were macroscopically unrecognizable and were diagnosed on the basis of microscopic findings. Pulmonary metastases of GIST are rare and account for only 2% of all metastases [1]. In this case, the GIST tumor cells flowed into general circulation and formed multiple metastatic lesions in the lung. Even if radical resection had been performed, micro-metastases may remain and become apparent after a long period of time.

Regarding recurrence of GIST-associated liver metastasis, it was reported that 90% of re-recurrences occur even after surgery, and the guidelines recommend chemotherapy [2, 14, 15]. In patients with GIST, the effect of imatinib differs depending on the type of mutation in the c-kit gene. Frolov et al. reported that the response rate in cases with Exon 11 and Exon 9 mutations was 83.5% and 47.8%, respectively [16]. In the present case, a mutation was found in Exon11, and therefore, a high effect of imatinib was expected. However, administration of imatinib caused fatal tumor hemorrhage, which impaired the possibility of continued treatment.

In this case, chemotherapy for up to 7 years was possible as it could maintain the SD for a long period of time. Sunitinib is usually administered for 4 weeks and then withdrawn for 2 weeks, whereas regorafenib is administered for 3 weeks and withdrawn for 1 week. However, in this case, both drugs were administered for 1 week and withdrawn for 2–4 weeks; therefore, chemotherapy could be continued without serious side effects. For slow-growing tumors, long-term chemotherapy with minimal side effects may contribute to long-term prognosis.

Tumor bleeding is a diagnostic hallmark of GIST. Gastrointestinal bleeding and intraperitoneal bleeding have been reported, and tumor bleeding also triggered the diagnosis in this case [17, 18]. In the present case, it could not be established with certainty whether chemotherapy (imatinib) was the only cause of the bleeding, or whether the bleeding would have occurred anyway because of nature of the tumor (GIST). In this case, tumor bleeding occurred twice within a short period (within 10 days) after administration of Imatinib, and no tumor bleeding was observed after discontinuation of imatinib. Therefore, imatinib was the most likely cause of the bleeding. We speculate that the tumor tissue had become fragile due to necrosis and that exposure to imatinib made it bleed easily. It is possible that tumor bleeding did not have occurred with sunitinib or regorafenib therapy, because they have a weaker antitumor effect than imatinib. Continued awareness of severe bleeding in GIST patients is critical.

The limitation of this study is that it is not possible to make a definitive diagnosis of metastasis of GIST due to the lack of pathological specimens 25 years ago.

Conclusively, GIST may recur long after surgery and its risk are not associated with conventional risk classification. The current guidelines recommend follow-up up to 10 years after surgery, but follow-up for more than 10 years is desirable. We would also like to emphasize the importance of adjuvant chemotherapy to prevent recurrence over a long time.

## Figures and Tables

**Table 1:** Reported cases of GIST recurrence occurring later than 10 years after surgery.

Author (year)	Reference number	Age	Sex	Primary site	Size (cm)	Mitotic index	Risk classification (Fletcher)	Recurrence site	Time to recurrence (years)
Ballarini (1998)	8	62	Male	Stomach	4	1/50 HPF	Low	Liver	11
Matsuoka (2003)	9	58	Male	Rectum	4	2/10 HPF	Intermediate	Liver	12
Nowain (2005)	10	39	Female	Small intestine	10	N/A	Intermediate	Liver	17
Matsuoka (2007)	11	55	Female	Small intestine	14	N/A	High	Liver	17
Ginori (2015)	12	71	Male	Duodenum	2.5	1/50 HPF	Low	Liver	29
Our case	—	56	Male	Small intestine	N/A	N/A	N/A	Liver	25

N/A: not available.

## Data Availability

The data used to support the findings of this study are available from the corresponding author upon request.
